# *ZNF300P1* Encodes a lincRNA that regulates cell polarity and is epigenetically silenced in type II epithelial ovarian cancer

**DOI:** 10.1186/1476-4598-13-3

**Published:** 2014-01-06

**Authors:** Brian Gloss, Kim Moran-Jones, Vita Lin, Maria Gonzalez, James Scurry, Neville F Hacker, Robert L Sutherland, Susan J Clark, Goli Samimi

**Affiliations:** 1 Kinghorn Cancer Centre and Cancer Research Program, Garvan Institute of Medical Research, 370 Victoria Street, 2010, Darlinghurst, NSW, Australia; 2 St. Vincent’s Clinical School, University of New South Wales, 2010, Sydney, NSW, Australia; 3 Hunter Area Pathology Service, John Hunter Hospital, 2310, New Lambton, NSW, Australia; 4 School of Women’s and Children’s Health, University of New South Wales, and Gynaecological Cancer Centre, Royal Hospital for Women, 2031, Sydney, NSW, Australia

**Keywords:** lincRNA, Ovarian cancer, Methylation, Epigenetics, *ZNF300P1*, *ZNF300*

## Abstract

**Background:**

We previously identified that the CpG island-associated promoter of the novel lincRNA *ZNF300P1* (also known as *LOC134466*) is frequently hypermethylated and silenced in ovarian cancer tissues. However, the function of *ZNF300P1* was unknown. In this report we demonstrate that *ZNF300P1* is involved in the regulation of key cell cycle and cell motility networks in human ovarian surface epithelial cells, and may play a role in promoting metastasis in ovarian cancer cells.

**Methods:**

We applied methylated DNA immunoprecipitation on whole genome promoter tiling arrays and Sequenom assays to examine methylation status of *ZNF300P1* in multiple ovarian cancer cell lines, as well as in normal ovarian and ovarian tumor tissues. Transcript profiling was used to investigate the effects of *ZNF300P1* suppression in ovarian cancer cells. We utilized siRNA knockdown in normal ovarian surface epithelial cells and performed cellular proliferation, migration and adhesion assays to validate and explore the profiling results.

**Results:**

We demonstrate that *ZNF300P1* is methylated in multiple ovarian cancer cell lines. Loss of *ZNF300P1* results in decreased cell proliferation and colony formation. In addition, knockdown of the *ZNF300P1* transcript results in aberrant and less persistent migration in wound healing assays due to a loss of cellular polarity. Using an *ex vivo* peritoneal adhesion assay, we also reveal a role for *ZNF300P1* in the attachment of ovarian cancer cells to peritoneal membranes, indicating a potential function of *ZNF300P1* expression in metastasis of ovarian cancer cells to sites within the peritoneal cavity.

**Conclusion:**

Our findings further support *ZNF300P1* as frequently methylated in ovarian cancer and reveal a novel function for *ZNF300P1* lincRNA expression in regulating cell polarity, motility, and adhesion and loss of expression may contribute to the metastatic potential of ovarian cancer cells.

## Background

Ovarian cancer is a heterogeneous disease of the female reproductive tract which, despite its relatively low incidence in developed countries, carries a poor prognosis. Despite advances in the detection and treatment of ovarian cancer, it remains the 5th leading cause of cancer death in women [1]. Epithelial ovarian cancer (EOC) comprises 90% of all ovarian cancer cases [2]. Type I EOC primarily consists of low-grade serous, mucinous, endometrioid and clear cell subtypes, and is characterized as slow growing with intact DNA repair machinery [3]. Type II EOC, also known as high-grade serous ovarian cancer, comprises 70% of EOC cases [4] and is characterized by rapid growth with no identified precursor lesions, and genome instability (p53 loss) [5]. The molecular events underlying Type II EOC remain poorly understood, and despite initial response to chemotherapy, these tumors often recur with chemo-resistance. EOC is typically diagnosed at late stage, when the tumor has spread beyond the pelvic region into the peritoneal cavity, making complete surgical removal extremely difficult. Despite recent advances in surgery and adjuvant chemotherapeutics, the overall five-year survival rate for EOC remains at only 40%, in part due to common and rapid peritoneal spread of disease, indicating the need to understand the genetic and epigenetic events underlying EOC progression.

We recently identified that the CpG island-associated promoter of *ZNF300P1*, a candidate long-intergenic non-coding (linc) RNA, was hypermethylated in EOC cell lines, and this was associated with loss of expression [6]. LincRNAs are polyadenylated RNA transcripts that are transcribed by RNA polymerase II and do not encode for protein, although they do carry epigenetic signatures similar to those found in protein-coding genes [7]. In cancer, recent studies have demonstrated a link between lincRNA expression and disease progression and outcome related to the role of lincRNAs in gene expression regulation [7,8].

*ZNF300P1*, also known as *LOC134466*, is a pseudogene of the human zinc finger protein ZNF300 (sharing 89% identity), and is characterized as a non-coding transcript. Khalil and colleagues identified *ZNF300P1* as a lincRNA using a computational algorithm that eliminates transcripts with protein-coding domains and chromatin signatures that reflect transcribed genes [9]. Our evaluation showed that *ZNF300P1* expression was also repressed in human EOC tissues compared to normal ovarian surface epithelial cells (OSE) [6]. Quantitative methylation analysis discriminated 27 EOC tumors from 14 normal OSE samples with a high degree of accuracy (81% sensitivity, 92% specificity [6]), suggesting its potential as an EOC biomarker. Finally, methylation-specific headloop-suppression PCR (MSH-PCR) screening of 159 high-grade EOC tumors demonstrated methylation of *ZNF300P1* in 81% of tested tumors, suggesting that it may serve a functional role in EOC [6]. Indeed, studies have demonstrated that lincRNAs, including *ZNF300P1*, are associated with Polycomb Repressive Complex (PRC2) and CoREST chromatin modifying complex proteins [9], supporting the notion that *ZNF300P1* may be involved in gene expression regulation and thus may serve a functional role in cancer and other processes.

The function of *ZNF300P1* is currently unknown. Since *ZNF300P1* is frequently and specifically methylated and down-regulated in EOC, we sought to examine its potential role in regulating cell behavior in EOC. Our findings suggest that *ZNF300P1* is frequently methylated in EOC and reveal a novel function for *ZNF300P1* in regulating cell polarity, motility, and adhesion.

## Results

### *ZNF300P1* is epigenetically repressed in ovarian cancer

**Figure 1:**
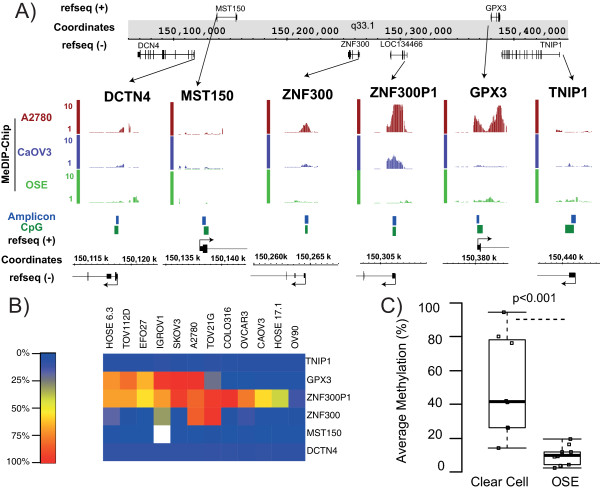
***ZNF300P1 *methylation patterns in EOC. (A)** MeDIP-Chip methylation tracks (vertical bars denote methylation score of 1–5) of A2780 (red) and CaOV3 (purple) EOC cell lines compared to grouped profiles of three OSE (green) mapped to chromosome 5q33.1. Features of the chromosomal region are also shown including strand specific genes (black), CpG islands (dark green) and the Sequenom assay designed to interrogate promoter methylation (blue). **(B)** Average promoter methylation (% methylation as heatmap) of CpG islands flanking *ZNF300P1* in EOC cell lines and immortalized OSE. Genes are sorted 5′ to 3′ on chromosome 5q33.1, cell lines are organized by similarity in methylation patterns. **(C)** Boxplot comparing average promoter methylation of *ZNF300P1* in the eight clear cell tumors compared to 19 OSE. The p-value is generated by a Mann–Whitney U test.

We have previously demonstrated long-range epigenetic silencing (LRES) of discrete genomic regions in colorectal and prostate cancer [10-12]. Regional repression is associated with DNA hypermethylation and/or chromatin remodeling of consecutive genes along the DNA strand. To investigate whether *ZNF300P1* methylation was embedded in a region of LRES in EOC, we evaluated methylated DNA immunoprecipitation on whole genome promoter tiling array (MeDIP-ChIP) profiles for normal ovarian surface epithelium (OSE) and A2780 and CaOV3 cancer cell lines as described ([6]) (Figure 1A). Evidence of hypermethylation at the CpG island associated with *ZNF300P1* was observed in both cancer cell lines, however hypermethylation of neighboring CpG islands (*ZNF300* and *GPX3*) was only observed in A2780 cells. The transcript profiles from the same study showed that this hypermethylation was indeed associated with repression of gene expression (Additional file 1: Figure S1A), suggesting that regional epigenetic silencing may occur near *ZNF300P1* in ovarian cancer. To investigate the frequency of surrounding CpG island methylation in further cell lines, Sequenom assays were designed to interrogate the methylation of five CpG islands associated with nearby genes *DCTN4*, *MST150*, *ZNF300*, *GPX3* and *TNIP1* genes. Methylation levels were evaluated for the five islands, as well as that for *ZNF300P1*, in 10 EOC cell lines and 2 immortalized OSE cell lines (Figure 1B). The CpG island for *GPX3*, located 3′ of *ZNF300P1*, exhibited methylation in 5/10 EOC cell lines and 1/2 OSE cell lines tested and the CpG island for *ZFN300*, located 5′ of *ZNF300P1*, was methylated in 3/10 EOC cell lines and 0/2 OSE cell lines. The largest region of neighboring CpG island methylation was observed in three cell lines: IGROV1, A2780 and TOV21G, with up to 150 kb of flanking methylation (*ZNF300*-*GPX3*). This genomic size is smaller than average for regions characteristic of LRES, which are typically megabases in scale [11]. Additionally, while one or two flanking CpG islands genes exhibited hypermethylation in up to 6 of 12 cell lines, none were as frequently methylated as *ZNF300P1* (10/10 EOC cell lines and 1/2 OSE) (Figure 1B). This result suggests that methylation of *ZNF300P1* is the key initiating event, rather than a part of LRES, in this region that can then spread in some cell lines [13]. We therefore performed a detailed analysis of methylation of *ZNF300P1* in primary ovarian tumors to determine its tendency in clinical EOC samples.

### *ZNF300P1* methylation is not limited to serous subtypes of EOC

While high-grade serous tumors comprise the majority of EOC, the less common and molecularly distinct clear cell subtype (5%) [14], if diagnosed at late stage, carries an extremely poor prognosis [15]. Therefore, to evaluate whether methylation was conserved in this histological subtype, *ZNF300P1* methylation was investigated in 8 clear cell tumor samples by Sequenom and compared to methylation in 19 OSE samples (Figure 1C). *ZNF300P1* hypermethylation (>50% average promoter methylation) was observed in 4 of the 8 clear cell tumors with methylation levels significantly higher in tumors than OSE (p-value <0.001). This result indicates that the frequency of *ZNF300P1* hypermethylation is comparable between clear cell and serous EOC [6], and significantly higher than in OSE. These findings suggest that methylation of *ZNF300P1* may not be a unique feature of Type II EOC.

### *ZNF300P1* repression impacts transcriptomic networks of immortalized ovarian epithelial cells.

Having originally identified *ZNF300P1* repression as a common feature of EOC and other cancer types, we sought to evaluate its biological impact in the immortalized normal ovarian cell line HOSE17.1, which is derived from normal ovarian surface epithelium and which expresses high levels of *ZNF300P1* and exhibits limited methylation [6]. As a candidate lincRNA, we hypothesized that a reduction of *ZNF300P1* expression, as seen in ovarian cancer cell lines (Additional file 1: Figure S1B), would recapitulate transcriptional network and the cell phenotype aberrations observed in cells harboring *ZNF300P1* DNA methylation. To test this, we analyzed RNA transcript profiles of HOSE17.1 cells following siRNA-mediated knockdown of *ZNF300P1* (HOSE17.1-siZP1). To ensure that expression of the highly homologous *ZNF300* is not affected upon knockdown of *ZNF300P1*, we compared expression of *ZNF300* in HOSE 17.1-siZP1 knockdown cells and non-targeting control knockdown cells (HOSE 17.1-siNTC) by both microarray and qPCR (Additional file 2: Figure S2A). Our results demonstrate that expression of *ZNF300* is not altered upon knockdown of *ZNF300P1*, suggesting that any gene expression changes detected in the knockdown cells are due specifically to repression of *ZNF300P1*. A total of 752 genes (509 up-regulated and 243 down-regulated) were identified that exhibited ≥1.3-fold (p-value < 0.01) differential expression upon knockdown of *ZNF300P1*. To validate our microarray analysis, we chose nine genes, based on statistical significance and functional annotation, for validation by qPCR (Additional file 2: Figure S2B). Most of these genes demonstrated differential expression by qPCR in knockdown cells compared with controls, confirming the microarray results.

Ingenuity Pathways Analysis^™^ (IPA) was utilized to identify gene networks and functions altered by down-regulation of *ZNF300P1* (Table 1). The top gene network affected by repression of *ZNF300P1*, Cell Cycle, DNA Replication, Recombination, and Repair, Cell-To-Cell Signaling and Interaction (score = 41, Table 1), is presented in Additional file 2: Figure S2C. The top disease network affected was cancer (186 molecules, p = 0.016, Table 1) and the top molecular function was cellular movement (80 molecules, p = 0.017, Table 1). These data implicate *ZNF300P1* repression in diverse molecular pathways commonly perturbed in cancer.

**Table 1:** **Ingenuity pathway analysis in cells by down-regulation of *ZNF300P1***

**Ingenuity pathway analysis: top functions**				
	**p-value**	**# molecules**		
Top Network: Cell Cycle, DNA Replication, Recombination, and Repair, Cell-To-Cell Signalling and Interaction				
Top Disease: Cancer	1.61E-02	186		
Top Molecular Function: Cellular Movement	1.68E-02	80		
Genes Up Regulated: KEGG Pathways				
	Count	%	Fold- Enrichment	corrected p-value
hsa04510: Focal adhesion	16	4.35	4.17	0.000
hsa04512: ECM-receptor interaction	8	2.17	4.99	0.047
Genes Down Regulated: KEGG Pathways				
	Count	%	Fold- Enrichment	corrected p-value
hsa04621: NOD-like receptor signalling pathway	7	3.23	7.97	0.022

To further investigate the nature of these molecular aberrations, functional annotation (DAVID) of gene ontology term (GO-term) enrichment and KEGG pathways associated with these expression changes were evaluated in the 752 genes de-regulated by *ZNF300P1* knock-down (Table 1). KEGG pathway analysis showed that the top canonical signaling pathways up regulated following *ZNF300P1* repression were focal adhesion, and the related ECM-receptor interactions pathway. Evidence of repression of the NOD-like pathway suggested that cell death pathways were being perturbed. Interestingly, GO-terms for cellular adhesion were enriched in the up-regulated gene list (complementing the KEGG analysis), whereas terms associated with cell cycle were enriched in the down-regulated gene list (Additional file 3: Table S1). These data suggested that *ZNF300P1* repression results in activation of cellular movement and repression of cell cycle processes.

### Loss of *ZNF300P1* is associated with phenotypic and cell behavior changes

**Figure 2:**
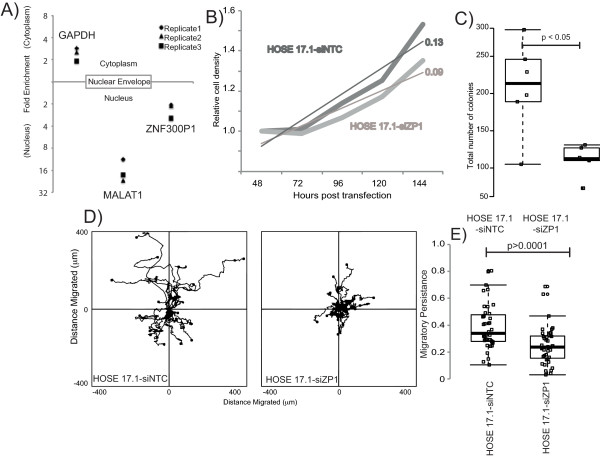
**ZNF300P1 cellular localization and phenotypic changes. (A)** MALAT1 and GAPDH are included as nuclear and cytoplasmic transcript controls, respectively. Data is presented as mean of 3 independent experiments ± S.D. The effect of *ZNF300P1* knockdown on HOSE17.1 cells. **(B)** Representative population growth of HOSE17.1 cells following transfection with non-targeting control (HOSE17.1-siNTC; dark grey) or siRNA against *ZNF300P1* (HOSE17.1-siZP1; light grey). A line of best fit is shown for both conditions. **(C)** Representative colony formation at 10 days post-transfection. Each data point represents total number of colonies per 4 wells. **(D)** Windrose plots of cell migration patterns following an *in vitro* wound healing assay. Cell starting position is designated as 0,0 and the path taken over 24 hours is measured every 10 minutes (μm). **(E)** Boxplot of persistent migration ratios for individual cells from a representative experiment. Persistence was defined as the ratio of the total path length divided by the Euclidean displacement. P-values are generated by a Mann–Whitney U test.

Transcriptome analysis indicated that *ZNF300P1* knockdown results in decreased expression of genes involved in cell cycling. We sought to determine the role that *ZNF300P1* might play on cancer cell processes including proliferation and motility. Our first step towards defining a functional role for *ZNF300P1* was to determine the cellular localization of the lincRNA in HOSE17.1 cells. As demonstrated in Figure 2A, our studies reveal that *ZNF300P1* is enriched in the nucleus, lending further support to a potential functional role in ovarian cancer.

We next evaluated cell proliferation and colony formation in normal HOSE 17.1 cells in which *ZNF300P1* expression was knocked-down (HOSE17.1-siZP1). Expression analysis of *ZNF300P1* transcript levels following siRNA treatment showed a maximal knockdown of ~50% over 72 hours post transfection with increased variation at 96–120 hours (Additional file 1: Figure S1C). Proliferation assays showed that *ZNF300P1* knockdown results in decreased cell growth, with HOSE17.1-siZP1 cells entering log-phase growth later relative to control (HOSE17.1-siNTC) cells (Figure 2B). These results were validated in colony formation assays, which showed that HOSE17.1-siZP1 cells had impaired colony formation (Figure 2C). However, HOSE17.1-siZP1 cells displayed no defect in cell cycle, by propidium iodide staining and flow cytometry (Additional file 4: Figure S3), nor were proliferation differences explained by increased cell numbers in the supernatant of the culture vessel (data not shown), indicating no obvious cell cycle defects nor altered sensitivity to anoikis. Together, these results show that the gene networks associated with the cell cycle altered following *ZNF300P1* knockdown manifest as a reduction in population growth, but show no evidence of cell-cycle blocks nor altered anoikis-related cell death.

**Figure 3:**
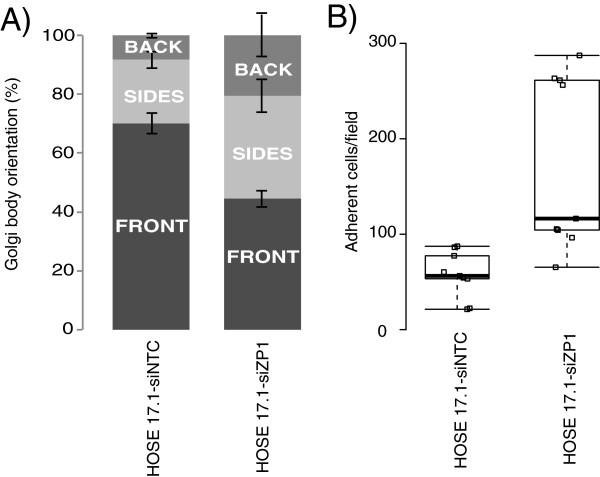
**Effect of *ZNF300P1 *knockdown on cell polarity and adhesion. (A)** Stacked barchart of Golgi body orientation relative to woundspace and the nucleus 6 hrs after wounding *in vitro*. Cells were visually scored as having Golgi in the front quarter of the cell (generally considered a polarised orientation), or the back quarter, or sides. Data is mean of 3 independent experiments ± S.D. **(B)** Boxplot of *ex vivo* peritoneum adhesion assays. The number of cells adhered to mouse peritoneum after 3 hrs was counted per field, data is mean of 3 independent experiments ± S.D. The p-value is generated by a Mann–Whitney U test.

IPA identified cellular movement as the top molecular function significantly altered by *ZNF300P1* knockdown (Table 1). We therefore next sought to investigate the effect of *ZNF300P1* knockdown on cell adhesion and motility in HOSE17.1 cells. Boyden chamber trans-well migration assays showed no clear difference in migration between HOSE17.1-siZP1 cells and HOSE17.1-siNTC cells (data not shown). However, live cell tracking of HOSE17.1-siZP1 and HOSE17.1-siNTC cells during wound healing showed a clear defect in persistent migration (defined as the total distance covered by the cell divided by the Euclidean distance covered) into the wound space by HOSE17.1-siZP1 cells (Figure 2D, E and Additional file 5: Movies 1 and 2). In order to test whether this defect was due to a loss in the cells’ ability to polarize the cytoskeleton relative to the wound space, immunofluorescence of Golgi orientation relative to the nucleus, cytoskeleton and wound space was quantified in HOSE17.1 cells 6 hours following wounding (Figure 3A). The results demonstrate a significant decrease in the number of cells with the Golgi arranged towards the directional front in HOSE17.1-siZP1 cells, consistent with a defect in cellular polarity in these cells.

A hallmark and major cause of mortality of EOC is the spread of cancer cells from the ovary to sites within the peritoneal cavity. We have shown that DNA methylation-associated repression of *ZNF300P1* occurs commonly in EOC, and that the repression of this lincRNA in epithelial ovarian cells results in a defect in cell polarity, which may contribute to metastasis. Thus, to further explore the potential influence of *ZNF300P1* on this crucial step in EOC progression, we tested the capacity of cells to adhere to mouse peritoneal tissue, using an *ex vivo* peritoneal adhesion assay. HOSE17.1 cells with repressed *ZNF300P1* expression demonstrated significantly increased peritoneal adhesion compared with HOSE17.1-siNTC cells (p-value <0.05 Mann–Whitney U-Test) (Figure 3B), implicating *ZNF300P1* loss, as commonly seen in EOC cases, in promoting adhesion of EOC cells to peritoneal surfaces, thus potentially a contributing factor to the metastatic spread of EOC.

## Discussion

We have previously demonstrated that methylation of the lincRNA *ZNF300P1* is a potential biomarker in EOC [6]. Here, we investigate the novel role that *ZNF300P1* repression may play in ovarian cancer development and progression, and show that lincRNA *ZNF300P1* plays a role in regulating cell polarity, and how loss of expression may contribute to the metastatic potential of ovarian cancer cells.

LincRNAs are gaining increasing importance both as biomarkers in cancer (HOTAIR: breast [8], liver [16], pancreas [17]; MALAT-1: NSCLC [18]; PCAT-1: prostate [7]), and as regulators of complex and diverse biological functions. Guttman *et al*. developed a screen to identify lincRNAs using histone methylation markers H3K4 and H3K36 to demarcate transcribed RNAs located outside of protein coding regions [19]. Khalil and colleagues applied this approach in human cells, and identified *ZNF300P1* as a lincRNA and used RIP-Chip to show its association with Polycomb Repressive Complex (PRC2) and CoREST chromatin modifying complex proteins, indicating the potential for *ZNF300P1* to be involved in modification of chromatin structure [9]. LincRNAs have also been proposed to alter transcriptional networks using four modes of action [20]. These are: 1) as decoys to titrate DNA-binding proteins (or miRNAs [21]); 2) as scaffolds to bring proteins together within a complex; 3) as guides to recruit proteins, such as chromatin modification complexes to DNA; or 4) as enhancers to bring distal portions of the genome into close proximity through looping. The transcriptional analyses undertaken within this study, using siRNA against *ZNF300P1*, indicate that loss of *ZNF300P1* expression, as seen resulting from hypermethylation in ovarian cancer samples, results in both up- and down- regulated gene expression (Additional file 3: Table S1). While the precise mechanisms by which these genes are transcriptionally altered are outside the scope of this study, it is possible that *ZNF300P1* suppression may alter transcriptional networks via modification of chromatin, guiding the repressive complex to genes/promoters to regulate their transcription.

However, a number of recent studies have also proposed that a sub-population of lincRNAs arise from pseudogenes [21,22], and act as endogenous competitors, altering the distribution of miRNA molecules on their targets [21]. *ZNF300P1*, also known as *LOC134466*, is a pseudogene of the human zinc finger protein ZNF300. Little is known of the function of this nucleus-restricted transcription factor [23]. Demonstrated to be up-regulated in cancer biopsies [24], ZNF300 binds the sequence C(t/a)GGGGG(g/c)G, found in the promoter regions of genes including IL-2, IL-2Rb, CD44, p53, TNF-a, and TRAF2, which play crucial roles in various tumorigenic and inflammatory processes. Furthermore, ZNF300 induces the NF-kB pathway, in turn inducing IL-6 and IL-8, potentially exacerbating inflammation and promoting tumor metastasis [24]. One potential means by which lincRNAs arising from pseudogenes have been proposed to function is by acting as a decoy for miRNAs targeting the protein-coding gene. Given that ZNF300 is up-regulated in cancer, and promotes inflammation and metastasis, the repression of *ZNF300P1* associated with methylation in ovarian cancer make it unlikely to be functioning as an miRNA decoy. Furthermore, our analysis of ZNF300 expression upon *ZNF300P1* silencing in HOSE17.1 cells showed no significant changes (Additional file 2: Figure S2A), indicating that it is unlikely that *ZNF300P1* acts as an endogenous decoy for miRNAs targeting *ZNF300*.

Notably, we have confirmed nuclear enrichment of *ZNF300P1* (Figure 2A) [23], and have shown that loss of *ZNF300P1* expression in ovarian cells results in the perturbation of several key pathways involved in cell cycle, cell movement, and cell-to-cell signaling and interaction (Table 1 and Additional file 2: Figure S2C). These results suggest that DNA hypermethylation of the promoter CpG island of *ZNF300P1* may play a key role in the malignant progression of ovarian cancer. While repression of the lincRNA clearly affected the ability of cells to proliferate and form colonies, it had no discernible effect on the cell cycle, indicating that *ZNF300P1* does not function as a classic tumor suppressor. Interestingly, *ZNF300P1* was recently identified as methylated in small cell lung cancer-derived cell lines [25] potentially indicating that loss of expression of this transcript may be common to several cancer types.

The major phenotypic outcomes we identified to be associated with *ZNF300P1* silencing was decreased polarity and the resultant loss of migratory persistence (Figures 2 and 3), both key in cancer development and the aberrant spread of cancer cells [26]. These phenotypes, together with the enhanced ability of HOSE17.1 cells lacking *ZNF300P1* to adhere to mouse peritoneum, may provide an advantage for cells to more efficiently colonize the peritoneal tissue, thus reducing the efficacy of surgical treatment in EOC.

## Conclusions

We have demonstrated that the lincRNA *ZNF300P1* is frequently hypermethylated and silenced in ovarian cancer. We have determined that suppression of *ZNF300P1* in ovarian cancer cells results in decreased proliferation and colony formation, as well as loss of cellular polarity. We also reveal a novel role for *ZNF300P1* in the adhesion of ovarian cancer cells to the peritoneal membrane, suggesting a potential function in ovarian cancer metastasis. Together, these results provide the first evidence of the downstream biological effects of methylation-mediated repression of *ZNF300P1* in ovarian cancer, and implicate the specific targeting of the transcript, and the resultant loss of expression, as a critical step in EOC progression.

## Methods

### Cell line, tissue and OSE collection and processing

Ten standard cancer cell lines derived from various subtypes of EOC: serous (SKOV3, OVCAR3, IGROV, OV90, COLO316, A2780 and CaOV3), endometrioid (TOV112D), clear cell (TOV21G) and mucinous (EFO27), as well as two human immortalized OSE cell lines (HOSE 6.3 and HOSE 17.1), were obtained and cultured as described previously [6,27]. Fresh-frozen tumor (FFT) samples were obtained with written informed consent from women undergoing debulking surgery for EOC at the Royal Hospital for Women (RHW, Sydney, Australia), snap frozen in liquid nitrogen and stored at −80°C. Clinico-pathological characteristics of patient samples are presented in Additional file 6: Table S2. Pathologically normal OSE cell samples were obtained with written informed consent, by brushing the ovary during surgery for non-ovarian gynecological malignancies followed by expansion of epithelial cells in culture. Cultures were evaluated for purity by staining for high molecular weight cytokeratin to exclude stromal contamination and maintained in culture as previously described [28]. Cell pellets from passage 3 or earlier were processed for DNA extraction. Experimental procedures were approved by the Human Research Ethics Committee of the Sydney South East Area Hospital Service Northern Section (00/115).

### Nucleic acid extraction and processing

Total RNA was extracted with Trizol (Invitrogen) or RNeasy mini kit (Qiagen). Genomic DNA was extracted from tumor and OSE with QiaAMP mini kits (Qiagen), from FFPE tissue with Gentra Puregene DNA isolation kit (Qiagen) and from cell lines with the Stratagene DNA extraction Kit (Agilent). 1–2 μg genomic DNA isolated from human blood (unmethylated control, Roche Applied Sciences), *in vitro* methylated DNA (Chemicon International) and RNase-treated cell and tumor DNA was bisulphite-converted either using the Epitect kit (Qiagen) or as previously described [29,30].

### Sequenom massARRAY quantitative methylation analysis

Sequenom methylation PCR assays were designed according to [31] to interrogate methylation levels at CpG islands immediately upstream (*DCTN4*, *MST150* and *ZNF300*) and downstream (*GPX3* and *TNIP1*) of *ZNF300P1*. Primers (Additional file 7: Table S3) were optimized for bisulphite-converted DNA specificity, and tested for bias using a thermal gradient on mixes of 50:50 methylated: unmethylated template. Triplicate PCR reactions were pooled, and applied to spectrochips according to manufacturer’s instructions for MALDI-TOF analysis (Sequenom). Results were analyzed using epityper software and RseqMeth [32] CpG methylation levels were averaged across the amplicon to determine average methylation.

### Methylation-specific headloop-suppression PCR assay (MSH-PCR)

Methylation specific headloop-suppression assays (MSH-PCR) were performed as previously described [6]. Briefly, triplicate MSH-PCRs were performed on bisulphite-converted DNA from FFPE tissue samples. By normalizing the signal to *SFN*, which is methylated in all samples [33], or bisulfite-converted genomic DNA (rDNA), relative methylation was calculated for all samples.

### Knockdown conditions and transfection of siRNA

Colloidal suspensions of 25 nM *ZNF300P1* (Ambion #n263234) or non-targeting control (NT-C; Ambion #4390483) siRNA in Lipofectamine 2000 and opti-MEM (Invitrogen) were prepared for transfecting HOSE17.1 cells according to Lipofectamine 2000 transfection protocol. Peak knockdown of ~50% was observed in HOSE17.1 cells by qPCR (Primer sequences, see Additional file 7: Table S3) at 48–72 hours post transfection (Additional file 1: Figure S1C).

### Transcript profiling

Biological triplicate RNA samples from 72 hours post transfection with 25 nM siRNA were submitted to the Ramaciotti Centre (University of New South Wales) for transcription, labeling and application to Affymetrix Human Gene 1.0ST mRNA transcript-profiling arrays.

### Data analysis

Array data were evaluated using the LIMMA package either in GenePattern (Broad Institute, Cambridge MA, USA) or in the R environment [34]. Briefly, array CEL files were normalized and background-corrected by the RMA method. Differential expression between probe-sets in triplicate control or *ZNF300P1* siRNA arrays was calculated using Bayesian linear models with stringency cut-offs of 1.3 fold up or down following knockdown, with an unadjusted p-value of <0.01. Evaluation of gene list properties was performed using Functional annotation (GO terms and KEGG pathways) and gene networks in Ingenuity Pathway Analysis^™^ software.

### Transcript localization

Nuclear and cytoplasmic RNA was isolated using Ambion’s PARIS kit, according to the manufacturer’s instructions. qRT-PCR was performed using MALAT1 and GAPDH as nuclear- and cytoplasmic-enriched controls, respectively. ZNF300 and ZNF300P1 RNA levels were determined in HOSE17.1 cells transfected with siRNAs either against ZNF300P1, or a control sequence.

### Expression quantitative real-time PCR (qPCR)

qPCR assays for 18 s rRNA (cat# Hs99999901_s1), GAPDH (cat# Hs99999905_m1),ZNF300P1 (cat# Hs00859547_m1), ZNF300 (cat# Hs04177113_m1) and MALAT1 (cat# Hs00273907_s1) were purchased from Applied Biosystems and utilized for measuring gene expression. SYBR green qPCR assays were designed in PrimerExpressTM (Life Technologies; sequences in Additional file 7: Table S3)

### Population growth curves

HOSE17.1 cells treated with *ZNF300P1* or NT-C siRNA were seeded at 2.5x10^3^ cells/cm^2^ (population growth assays) or 600 cells/cm^2^ (colony formation). At indicated times, cells were fixed and stained using Diff Quick (Lab Aids). For quantitation, 10% acetic acid was used to destain the plates, and absorption measured at 595 nm.

### Wound healing: polarity assay and live cell tracking

Transfected cells were plated either onto glass coverslips (polarity assays), or into 6/12 well plates (live cell tracking), to reach confluence at 48 hrs post transfection. Using a 20 μl pipette tip, a wound was made in each monolayer, cells rinsed twice with medium, and incubated for 6 hrs (polarity assay) at 37°C, or into the heated chamber of a Zeiss Inverted microscope (live cell tracking). For polarity assays, cells were fixed using 4% PFA, and Golgi were stained using an anti-gm130 antibody (BD Biosciences, cat# 610823, 1/100 dilution), followed by alexa fluor-conjugated secondary, and counter-stained using phalloidin and DAPI. Slides were imaged using AxioVision (version 4.7) software, and Golgi orientation (relative to nucleus and wound space) determined using ImageJ software. For time-lapse photography, cells were imaged at 10 minute intervals over 24 hours by a Zeiss Axiovert 200 M inverted microscope and Image J software was used to track cell nuclei.

### *Ex vivo* peritoneal adherence assay

Ovarian cancer cell peritoneal adhesion was determined using an *ex vivo* assay, modified from previous studies [35]. Briefly, the peritoneal tissue was excised from euthanized 10–12 wk female Balb/c mice, divided along the midline into two pieces and placed into serum-free media. Syto9-labeled cells (1x10^5^) under each transfection condition were added to 96-well plates and peritoneal tissue laid over the wells, mesothelial surface down. The tissue was then covered by a glass coverslip and the inverted plate was incubated for 3 hrs at 37°C. The peritoneal tissue was then washed with serum-free medium, and attached cells observed and imaged using a Leica MZ16FA fluorescent dissection microscope, attached to a Leica DFC420C camera. Image J was used to count 6–9 fields per well.

## Competing interests

The authors declare that they have no competing interests.

## Authors’ contributions

BG and KM-J designed and performed the experiments; VL provided technical support; MG developed the normal cell lines; JS provided pathology expertise; NFH provided clinical samples and expertise; RLF was Head of the Cancer Program and originated the clinical biobank; SJC and GS conceived the idea and supervised the project. All authors have read and approved the final manuscript.

## Authors’ information

Goli Samimi and Susan J. Clark are equal senior authors.

## Supplementary Material

Additional file 1: Figure S1 (A) Raw expression values of genes associated with CpG islands flanking *ZNF300P1*. Data is from Affymetrix HGU133 Plus 2 transcript profiles for A2780 and CaOV3 cancer cell lines [6]. (B) Comparison of repression vs methylation in OSE cells (adapted from [6]). (C) The effect of *ZNF300P1* knockdown by siRNA on transcript levels relative to non-targeting control. Data is mean of 3 independent experiments ± S.D. Click here for file

Additional file 2: Figure S2 (A) *ZNF300* and *ZNF300P1* expression by microarray and qPCR following *ZNF300P1* knockdown by siRNA, relative to non-targeting control. Data is mean of 3 or 4 independent experiments ± S.D. *p-value < 0.05 by student’s t-test. (B) qPCR validation of selected differentially expressed genes in HOSE17.1-siZP1 *vs* siNTC. Expression values are relative to GAPDH and error bars represent the average ± SD of 3 replicate experiments. (C) Top IPA^™^ gene network perturbed by *ZNF300P1* repression in HOSE17.1 cells. Relative fold-change for each gene is shown by color (red indicates down-regulated and green represents up-regulated genes) following knockdown. *duplicate probe-set expression score is reconciled. Click here for file

Additional file 3: Table S1 Gene Ontology (GO) Process in cells by down-regulation of *ZNF300P1*. Click here for file

Additional file 4: Figure S3 Propidium iodide (PI) staining showing proportion of cells in stages of the cell cycle with relative proportions of populations over 3 replicate experiments ± SD. Click here for file

Additional file 5 **Movies 1 and 2: HOSE17.1 cells transfected with either siNTC (Movie 1) or siZP1 (Movie 2) were grown to confluence, 72 hours post transfection.** A wound space was made using a 20μL pipette tip, cells washed twice with fresh media, and then the plate transferred to the incubator chamber of the microscope. Imaging was performed from approximately 5 hours post wounding, and frames captured every 10 minutes, over a 24 hour period. Movies were generated from these images, and play at 20 frames/second. Click here for file

Additional file 6: Table S2 Clinico-pathological features of clinical samples assayed in this study. Click here for file

Additional file 7: Table S3 Primer Sequences for qPCR assays used in this study. Click here for file
