# Supplementary figures and images for: ZNF300P1 Encodes a lincRNA that regulates cell polarity and is epigenetically silenced in type II epithelial ovarian cancer
Source: Mol Cancer. 2014 Jan 6;13:3. doi: 10.1186/1476-4598-13-3 (PMC3895665; doi:10.1186/1476-4598-13-3)

A)

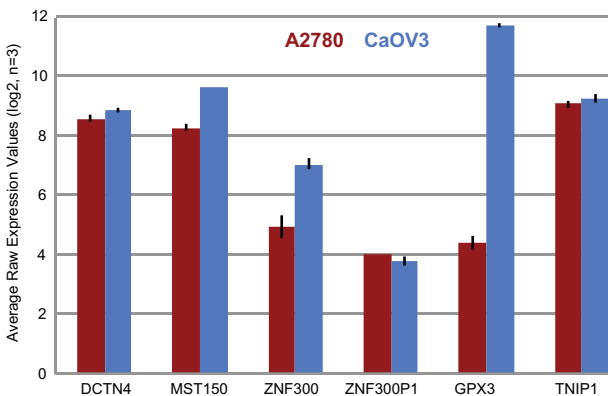

B)

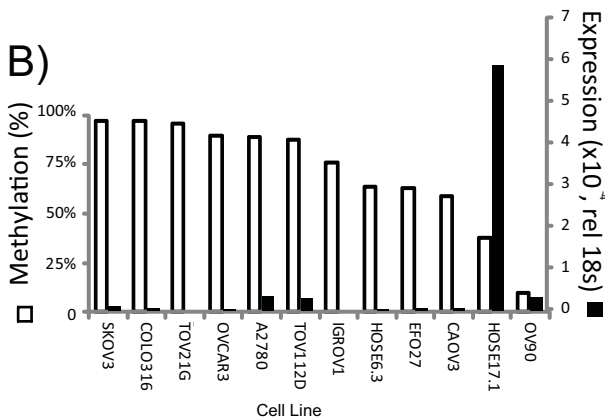

C)

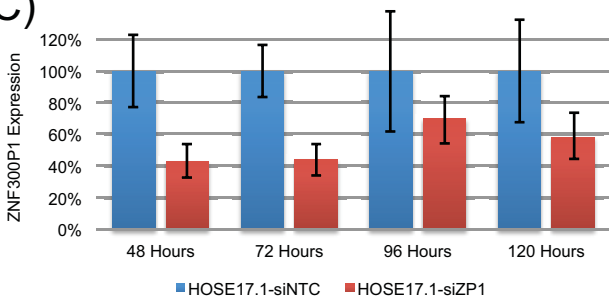

Supplementary Figure 1

Supplement: Additional file 1: Figure S1 — (A) Raw expression values of genes associated with CpG islands flanking ZNF300P1. Data is from Affymetrix HGU133 Plus 2 transcript profiles for A2780 and CaOV3 cancer cell lines [6]. (B) Comparison of repression vs methylation in OSE cells (adapted from [6]). (C) The effect of ZNF300P1 knockdown by siRNA on transcript levels relative to non-targeting control. Data is mean of 3 independent experiments ± S.D. [file 1476-4598-13-3-S1.pdf]

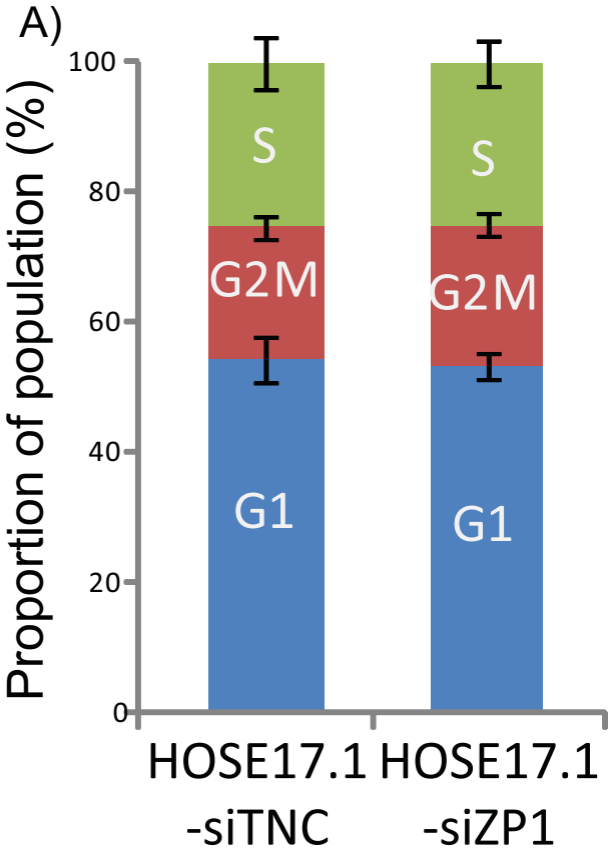

**Supplementary Figure 3**

Supplement: Additional file 4: Figure S3 — Propidium iodide (PI) staining showing proportion of cells in stages of the cell cycle with relative proportions of populations over 3 replicate experiments ± SD. [file 1476-4598-13-3-S4.pdf]
