# Supplementary material for: ZNF300P1 Encodes a lincRNA that regulates cell polarity and is epigenetically silenced in type II epithelial ovarian cancer
Source: Mol Cancer. 2014 Jan 6;13:3. doi: 10.1186/1476-4598-13-3 (PMC3895665; doi:10.1186/1476-4598-13-3)
Supplement: Additional file 2: Figure S2 — (A) ZNF300 and ZNF300P1 expression by microarray and qPCR following ZNF300P1 knockdown by siRNA, relative to non-targeting control. Data is mean of 3 or 4 independent experiments ± S.D. *p-value < 0.05 by student’s t-test. (B) qPCR validation of selected differentially expressed genes in HOSE17.1-siZP1 vs siNTC. Expression values are relative to GAPDH and error bars represent the average ± SD of 3 replicate experiments. (C) Top IPA™ gene network perturbed by ZNF300P1 repression in HOSE17.1 cells. Relative fold-change for each gene is shown by color (red indicates down-regulated and green represents up-regulated genes) following knockdown. *duplicate probe-set expression score is reconciled. [file 1476-4598-13-3-S2.pdf]

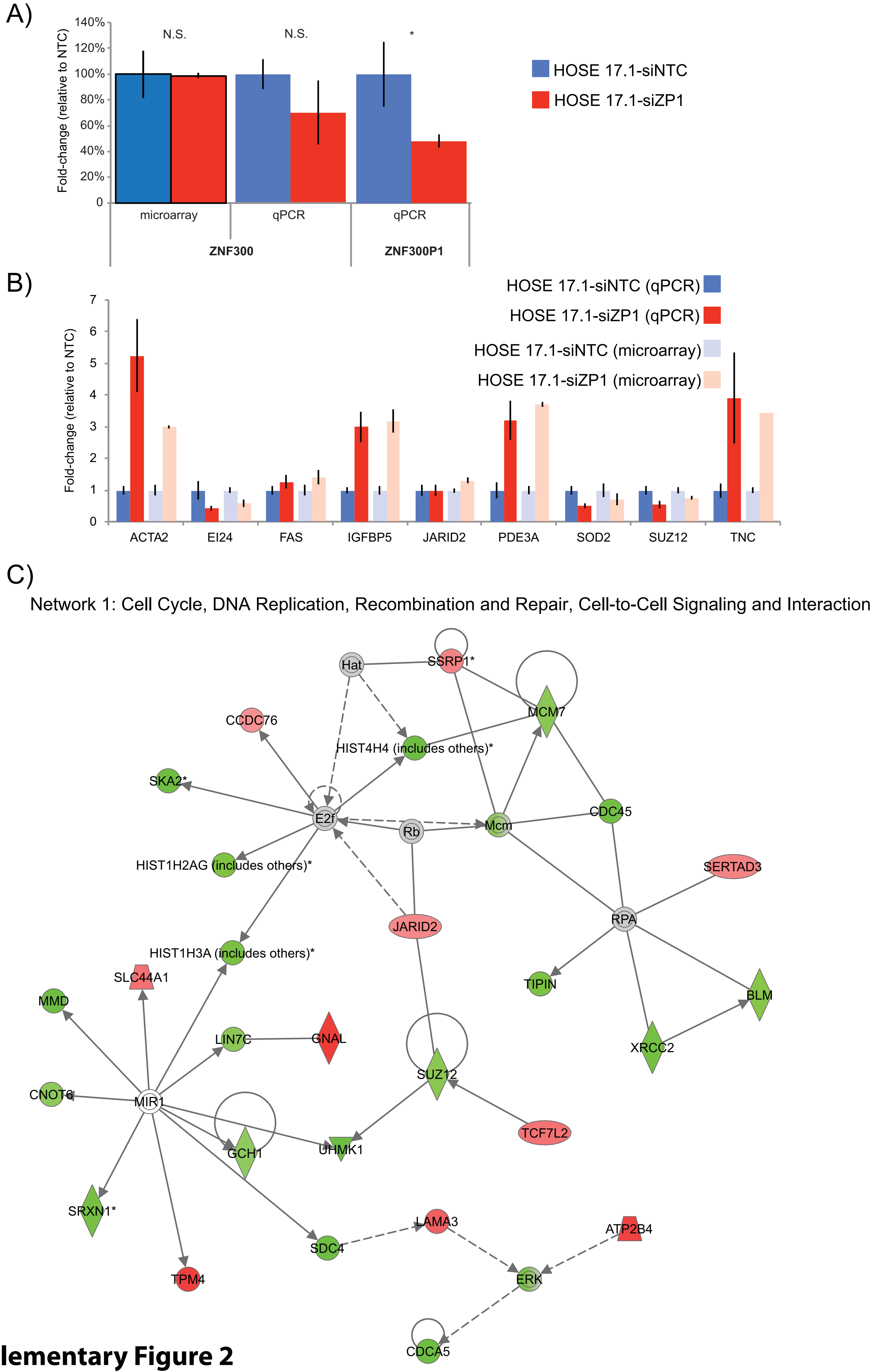

Supplementary Figure 2
