# Supplementary material for: ZNF300P1 Encodes a lincRNA that regulates cell polarity and is epigenetically silenced in type II epithelial ovarian cancer
Source: Mol Cancer. 2014 Jan 6;13:3. doi: 10.1186/1476-4598-13-3 (PMC3895665; doi:10.1186/1476-4598-13-3)
Supplement: Additional file 3: Table S1 — Gene Ontology (GO) Process in cells by down-regulation of ZNF300P1. [file 1476-4598-13-3-S3.docx]

| **Supplementary Table 1. Gene Ontology (GO) Process in cells by down-regulation of *LOC134466*** | | | | |
| --- | --- | --- | --- | --- |
| Genes Down Regulated: GO Biological Process | | | | |
|  | Count | % | Fold- Enrichment | corrected  p-value |
| GO:0007049~cell cycle | 29 | 13.36 | 3.34 | 0.000 |
| GO:0022402~cell cycle process | 24 | 11.06 | 3.80 | 0.000 |
| GO:0000279~M phase | 16 | 7.37 | 4.34 | 0.002 |
| GO:0022403~cell cycle phase | 18 | 8.29 | 3.88 | 0.002 |
| GO:0007067~mitosis | 13 | 5.99 | 5.28 | 0.002 |
| GO:0000280~nuclear division | 13 | 5.99 | 5.28 | 0.002 |
| GO:0000087~M phase of mitotic cell cycle | 13 | 5.99 | 5.19 | 0.002 |
| GO:0048285~organelle fission | 13 | 5.99 | 5.07 | 0.002 |
| GO:0000278~mitotic cell cycle | 16 | 7.37 | 3.86 | 0.003 |
| GO:0051301~cell division | 14 | 6.45 | 4.24 | 0.004 |
| GO:0051276~chromosome organization | 17 | 7.83 | 3.13 | 0.015 |
| GO:0006996~organelle organization | 30 | 13.82 | 2.01 | 0.043 |
|  |  |  |  |  |
| Genes Up Regulated: GO Biological Process | | | | |
|  | Count | % | Fold- Enrichment | corrected  p-value |
| GO:0032501~multicellular organismal process | 99 | 26.90 | 1.43 | 0.017 |
| GO:0032502~developmental process | 78 | 21.20 | 1.53 | 0.017 |
| GO:0030201~heparan sulfate proteoglycan metabolic process | 5 | 1.36 | 22.01 | 0.019 |
| GO:0048731~system development | 63 | 17.12 | 1.67 | 0.023 |
| GO:0022610~biological adhesion | 26 | 7.07 | 2.29 | 0.031 |
| GO:0007275~multicellular organismal development | 71 | 19.29 | 1.53 | 0.032 |
| GO:0060348~bone development | 10 | 2.72 | 5.01 | 0.035 |
| GO:0007155~cell adhesion | 26 | 7.07 | 2.29 | 0.039 |
| GO:0048856~anatomical structure development | 67 | 18.21 | 1.63 | 0.041 |
