# Supplementary material for: ZNF300P1 Encodes a lincRNA that regulates cell polarity and is epigenetically silenced in type II epithelial ovarian cancer
Source: Mol Cancer. 2014 Jan 6;13:3. doi: 10.1186/1476-4598-13-3 (PMC3895665; doi:10.1186/1476-4598-13-3)
Supplement: Additional file 6: Table S2 — Clinico-pathological features of clinical samples assayed in this study. [file 1476-4598-13-3-S6.docx]

| **Supplementary Table 2. Patient Characteristics** | |
| --- | --- |
| Ovarian Clear Cell Cancer cases (N=8) | |
| Mean Age (range) | 56 (47-70) |
| Stage | 1 (N=6)  2 (N=0)  3 (N=2)  4 (N=0) |
