# Supplementary material for: ZNF300P1 Encodes a lincRNA that regulates cell polarity and is epigenetically silenced in type II epithelial ovarian cancer
Source: Mol Cancer. 2014 Jan 6;13:3. doi: 10.1186/1476-4598-13-3 (PMC3895665; doi:10.1186/1476-4598-13-3)
Supplement: Additional file 7: Table S3 — Primer Sequences for qPCR assays used in this study. [file 1476-4598-13-3-S7.docx]

| **Supplementary Table 3. Primer Sequences** | |
| --- | --- |
| **Name** | **Sequence** |
| SFN_F | ATTGAGTAGAAAAGTAAYGAGGAGGGT |
| SFN_R | GTGTGTGATATTGTGCTTAATAAAATAACTATCCAACAAACCCAA |
| SFN_Probe2 | VIC-TGAAAGGCGTCGTGGAG -NFQ |
| ZNF300P1_F | AGTACGGGGTCGTTATTTTGAGATTTT |
| ZNF300P1_R | CGGAATGTGGATCCCTTCICAATCACTATAATACAA |
| ZNF300P1_ Probe | VIC-TCAACGCCTAACGAAT-NFQ |
| ACTA2-F1 | TGAGCGTGGCTATTCCTTCGT |
| ACTA2-R1 | CCCATCAGGCAACTCGTAACTC |
| EI24-F1 | TTGGTGGTGAAGAGATGGCTG |
| EI24-R1 | CTCTTTTGCTGGATTCGAGCAT |
| FAS-F1 | TTTCACTTCGGAGGATTGCTCA |
| FAS-R1 | TGGACGATAATCTAGCAACAGACG |
| IGFBP5-F1 | GAGCTACCGCGAGCAAGTCA |
| IGFBP5-R1 | GGGAGTAGGTCTCCTCGGCC |
| JARID2-F1 | CAACGATGAGATGCGGTTTGT |
| JARID2-R1 | GCCCATCTCATTAATCAGCCG |
| PDE3A-F1 | AGAGTGCCCCAGACCTATCCC |
| PDE3A-R1 | TCTACTTGGTGTCCGAGTGGCTA |
| SOD2-F1 | TTGGTGTCCAAGGCTCAGGTT |
| SOD2-R1 | ACACATCAATCCCCAGCAGTG |
| SUZ12-F1 | GCCAAACCTCTTGCCACTAGAAAT |
| SUZ12-R1 | CAGAGTACACCAAGGGCAATGC |
| TNC-F1 | GAAGCCTGACACTGAGTACGAGGT |
| TNC-R1 | TGTCAATAGCTGCCTTGCCATT |
